# Coupled DNA-labeling and sequencing approach enables the detection of viable-but-non-culturable *Vibrio* spp. in irrigation water sources in the Chesapeake Bay watershed

**DOI:** 10.1186/s40793-021-00382-1

**Published:** 2021-06-22

**Authors:** Leena Malayil, Suhana Chattopadhyay, Emmanuel F. Mongodin, Amy R. Sapkota

**Affiliations:** 1 Maryland Institute for Applied Environmental Health, University of Maryland School of Public Health, College Park, MD USA; 2 Institute for Genome Sciences, University of Maryland School of Medicine, Baltimore, MD USA

**Keywords:** Viable-but-nonculturable, *Vibrio*, DNA-labeling, Sequencing, Reclaimed water, Brackish water, BrdU

## Abstract

Nontraditional irrigation water sources (e.g., recycled water, brackish water) may harbor human pathogens, including *Vibrio* spp., that could be present in a viable-but-nonculturable (VBNC) state, stymieing current culture-based detection methods. To overcome this challenge, we coupled 5-bromo-2′-deoxyuridine (BrdU) labeling, enrichment techniques, and 16S rRNA sequencing to identify metabolically-active *Vibrio* spp*.* in nontraditional irrigation water (recycled water, pond water, non-tidal freshwater, and tidal brackish water). Our coupled BrdU-labeling and sequencing approach revealed the presence of metabolically-active *Vibrio* spp. at all sampling sites. Whereas, the culture-based method only detected vibrios at three of the four sites. We observed the presence of *V. cholerae*, *V. vulnificus*, and *V. parahaemolyticus* using both methods, while *V. aesturianus* and *V. shilonii* were detected only through our labeling/sequencing approach. Multiple other pathogens of concern to human health were also identified through our labeling/sequencing approach including *P. shigelloides*, *B. cereus* and *E. cloacae*. Most importantly, 16S rRNA sequencing of BrdU-labeled samples resulted in *Vibrio* spp. detection even when our culture-based methods resulted in negative detection. This suggests that our novel approach can effectively detect metabolically-active *Vibrio* spp. that may have been present in a VBNC state, refining our understanding of the prevalence of vibrios in nontraditional irrigation waters.

**Supplementary Information:**

The online version contains supplementary material available at 10.1186/s40793-021-00382-1.

## Introduction

As global freshwater resources are rapidly being depleted—due to population growth, climate change, over pumping of aquifers and other factors—states and nations are relying more heavily on nontraditional irrigation water sources (e.g., recycled water, brackish water) to ensure agricultural water security and prevent food insecurity [10, 67]. In some semi-arid and arid regions of the world, brackish water is the only remaining irrigation water source available to farmers [29, 50]. The United States Geological Survey (USGS) defines brackish waters as having a dissolved-solids concentration between 1000 and 10,000 mg/L, which is greater than that of freshwater (> 1000 mg/L), but less than that of seawater (35,000 mg/L) [71]. In the semi-arid and arid regions of the United States and other countries, brackish water use has been largely restricted to relatively salt tolerant crops including cotton, sugar beets, barley, wheat, safflower, sorghum, soybeans and tomatoes [29, 32, 57, 58, 64]. The effects of salt stress on plants when irrigated with brackish water are well described in the literature [27, 70], and multiple mitigation strategies are being explored to enable these water sources to be suitable for irrigation purposes [32, 57, 58, 64].

Besides salinity, brackish water sources are known to harbor important human pathogens. *Vibrio* spp., for instance, are natural inhabitants of brackish estuaries such as the Chesapeake Bay, and include the following frank pathogens: *V. cholerae*, *V. parahaemolyticus* and *V. vulnificus* [14, 15, 81, 83]. Additionally, *Vibrio* spp. have been recovered from surface waters, such as rivers, creeks, and irrigation canals [37, 52, 65], as well as reclaimed water [25, 52, 53]. Human *Vibrio* infections can occur among people consuming raw or undercooked shellfish and among those working or recreating in contaminated waters [14, 15, 17, 56]. If *Vibrio*-contaminated water is also used to irrigate food crops that are eaten raw, this practice could represent an additional exposure pathway for human *Vibrio* infections [28, 76].

Hence, there is a need to further our understanding of the prevalence of *Vibrio* spp. in nontraditional irrigation water sources. Nevertheless, previous studies have provided evidence that *Vibrio* spp. can enter a viable-but-non-culturable (VBNC) state [3, 13, 36, 49, 55], limiting the ability of traditional culture methods to assess the true prevalence of these microorganisms in water bodies. On the other hand, the use of culture-independent, DNA-based techniques such as PCR and sequencing alone do not provide information on the viability of detected vibrios in water sources, since DNA detected through these methods can be derived from either dead or live organisms [40, 43, 84]*.*

This challenge can be addressed by using RNA-based sequencing approaches instead of DNA-based methods, particularly those targeting mRNA (which is only produced by metabolically-active cells), thus indicating the presence of live cells [1]. However, use of environmental RNA has received little attention mainly due to the observation that the persistence of RNA outside the organism is short-lived [60]. This notion has been challenged by Cristescu [16], who provided evidence that RNA may be abundantly and sufficiently present in the environment to evaluate the presence of live organisms [16]. Another major issue, however, is that high quality RNA extraction is more challenging than DNA extraction due to the rapid degradation of RNA which can occur because of inadequate sample processing and/or storage, or contamination with RNA degrading enzymes like RNases [73].

An alternative to RNA-based sequencing methods for the detection of live or metabolically-active bacteria includes the use of DNA labels such as 5-bromo-2’deoxyuridine, (BrdU). BrdU is a synthetic thymidine analog that incorporates into replicating DNA; therefore, bacteria detected in BrdU-treated samples are interpreted to be metabolically-active, viable members of the tested bacterial community [77]. This BrdU labeling technique has been used to identify the metabolically-active fraction of bacteria present in aquatic and soil environments [44, 75, 77]. Thus, the goal of this study was to evaluate whether coupling 5-bromo-2′-deoxyuridine- (BrdU) labeling with next-generation sequencing methods could enable the detection of VBNC vibrios*,* as well as the differentiation between metabolically-active and dead organisms in nontraditional irrigation water sources.

## Materials and methods

### Sampling sites and sample collection

Existing sampling sites characterized by CONSERVE: A Center of Excellence at the Nexus of Sustainable Water Reuse, Food and Health (www.conservewaterforfood.org) were leveraged for this study: one tidal brackish water river, one non-tidal freshwater creek, one agricultural pond and one water reclamation facility. Preliminary biweekly bacterial monitoring data from these sites for the period of September 2016 to September 2017 (data not shown) revealed the presence of *Vibrio* spp. Hence, 4 L grab samples from each site were subsequently collected over the course of 5 months (May 2018 to September 2018) (total *n* = 30 grab samples) to further characterize the presence of *Vibrio spp*. via culture-dependent and -independent methods.

Additionally, throughout our sampling period, the following water quality parameters were measured in triplicate using a ProDSS digital sampling system (YSI, Yellow Springs, OH, USA): water temperature (°C), conductivity (SPC uS/cm), pH, dissolved oxygen (%), oxidation/reduction potential (mV), turbidity (FNU), nitrate (mg/L), and chloride (mg/L). Precipitation (inches) data within the last 14 days were also obtained from Weather Underground (https://www.wunderground.com/).

### Sample processing

All samples were subjected to both BrdU labeling (1500 mL) and non-labeling (control subsamples, 1500 mL). Our sample processing method is summarized in Supplementary Figure 2.

#### Non-BrdU labeled water samples

Three 500 mL aliquots of each water sample were filtered through a 0.2 μM filter and then subjected to one of the following: 1) enrichment with alkaline peptone water (APW) (30 mL); 2) enrichment with estuarine peptone water (EPW) (30 mL); or 3) no enrichment (control sample). These samples were incubated for 24 h in the dark at room temperature.

#### BrdU labeled water samples

Three separate 500 mL aliquots of each water sample were subjected to BrdU treatment using our previously published method [44]. Briefly, 100 μL of 100 mM BrdU was added per 500 mL of water and incubated for 24 h in the dark at room temperature along with the non-BrdU labeled samples. No additional nutrients or carbon sources were added to the samples in order to maintain the original water conditions as much as possible. After incubation, each 500 mL BrdU-labeled water sample was filtered through a 0.2 μM filter and subjected to one of the following: 1) enrichment in APW; 2) enrichment in EPW; or 3) no enrichment (control sample).

#### Sample incubation and cultivation

All enrichments (BrdU labeled or not) and non-enriched control samples were incubated at 30 °C for 18–20 h. A loopful of growth from the enriched water samples (non-BrdU treated) was then streaked onto thiosulfate-citrate-bile salts-sucrose (TCBS) agar and incubated for 16–24 h at 35 °C. All colonies presenting as yellow (sucrose positive) or green (sucrose negative) on TCBS were selected and subjected to three rounds of streaking for purification and isolation. DNA of resulting purified isolates was then extracted using a heat shock method, which involves isolates being exposed to 100 °C heat and then transferred to ice.

### Multiplex PCR detection of Vibrio genus

To detect five pathogenic *Vibrio* species, a multiplex PCR amplification of the heat shocked isolates was performed following a published protocol [38]. The amplified products were then viewed via gel electrophoresis.

### DNA extraction

DNA extractions on all enriched and non-enriched BrdU-labeled and non-labeled water samples were performed using protocols previously published by our group [11, 12]. Briefly, 1 mL of PBS was added to lysing matrix B tubes (MP Biomedicals, Solon, OH, USA) containing: 1) filters (non-enriched samples); or 2) cells that were pelleted by centrifuging at 2450 x *g* for 20 min (enriched samples). Then, enzymatic cocktails containing lysozyme, mutanolysin, proteinase K and lysostaphin were added to the tubes and the tubes were incubated, after which the cells were mechanically lysed at 6.0 m/s for 40 s using an MP Biomedical FastPrep 24 (Santa Ana, CA, USA). The DNA was then purified using the Qiagen QIAmp DNA mini kit (Germantown, MA, USA) per the manufacturer’s protocol.

### Immunocapture of BrdU-treated samples

Immunocapture and isolation of BrdU-labeled were performed using our previously published methods [44]. Briefly, sheared and denatured herring sperm DNA (HS DNA) and monoclonal anti-BrdU (α-BrdU) antibody was mixed in a 1:9 ratio and incubated for 1 h at room temperature. Then DNA extracted from BrdU samples was denatured by heating for 5 mins at 100 °C and transferred onto ice for 5 mins. To this denatured sample DNA, HS DNA/ (α-BrdU) antibody complex was added and incubated for 1 h in the dark at room temperature with agitation to form DNA/HS DNA/ (α-BrdU) antibody complexes. Meanwhile, magnetic beads (Dynabeads, Dynal Inc., Invitrogen by Thermofisher Scientific) coated with goat anti-mouse immunoglobulin G were washed three times with 1 mg/ml acetylated bovine serum albumin (BSA) in phosphate-buffered saline (PBS) using a magnetic particle concentrator. The washed Dynabeads were then added to the DNA/HS DNA/ *α*-BrdU antibody complexes and in- cubated for an additional 1 h in the dark at room temperature. After incubation, the samples were washed in 0.5 ml PBS-BSA, and the BrdU-containing DNA fraction was eluted by adding 1.7 mM BrdU (in PBS-BSA) and incubating for 1 h in the dark at room temperature.

### 16S rRNA gene amplification and sequencing

Extracted DNA was then PCR amplified for the V3-V4 hypervariable region of the 16S rRNA gene using the universal primers 319F (ACTCCTACGGGAGGCAGCAG) and 806R (GGACTACHVGGGTWTCTAAT), and sequenced on an Illumina HiSeq2500 (Illumina, San Diego, CA) using a method developed at the Institute for Genome Sciences [21] and described previously [11, 12].

### 16S rRNA sequencing analysis

Following sequencing, 16S rRNA paired-end read pairs were assembled using PANDAseq [45], de-multiplexed, trimmed of artificial barcodes and primers, and assessed for chimeras using UCHIME in de novo mode implemented in Quantitative Insights Into Microbial Ecology (QIIME; release v.1.9.1) [7]. Quality trimmed sequences were then clustered de novo into Operational Taxonomic Units (OTUs) and taxonomic assignments were performed using VSEARCH [66] with a minimum confidence threshold of 0.97. The SILVA 16S database [62] in QIIME [7] was used for taxonomic assignments. Downstream data analysis and visualization were completed in RStudio (v.1.1.423) using R packages: biomformat (v.1.2.0) [48] vegan (v.2.4–5) [54], ggplot2 (v.3.1.0) [82], phyloseq (v.1.19.1) [47], and metagenomeSeq (v.1.16.0) [59]. When appropriate, data were normalized with metagenomeSeq’s cumulative sum scaling (CSS) [59] to account for uneven sampling depth. Prior to normalization, alpha diversity was measured using both the Observed richness metric and the Shannon diversity index [69]. Bray-Curtis dissimilarity was used for calculating beta diversity and was compared using analysis of similarities (ANOSIM) on normalized data (999 permutations).

## Results

### Water quality characteristics

Water quality characteristics of the four sampling sites (non-tidal freshwater creek, reclaimed water, tidal brackish river and freshwater pond) are shown in Table 1. Overall, ambient temperatures, irrespective of sampling site, increased from May to September 2018. Conductivity, nitrate and chloride levels were higher in the tidal brackish creek compared to the other sampling sites, and pH ranged from slightly acidic to slightly basic across all water sample types. Dissolved oxygen was higher in the freshwater pond compared to all other sampling sites.

**Table 1:** Water quality characteristic averages by sampling site, throughout the sampling period

Sampling sites	Sampling months	Precipitation (14d)	Water temp. (C)	DO%	Conductivity (SPC uS/cm)	pH	ORP mV	Turbidity (FNU)	Nitrate (mg/L)	Chloride (mg/L)
**Non-tidal freshwater**	May A	1.79	15.7	97.5	211.4	7.28	150.8	2.8	0.53	11.74
	May B	4.65	17.5	93.5	141.8	6.93	144.5	13.5	1.1	1.36
	Jun A	4.03	16.45	92.8	202.65	7.07	114.25	11.1	0.93	11.24
	Jun B	1.43	20.15	94.9	164	7.05	105.1	5.3	0.885	1.34
	Jul	0	22.65	95.5	204.7	7.29	87.97	0.2	0.23	0.09
	Aug	4.51	22.15	93	162.8	7.22	80.2	5.47	0.38	0.64
	Aug B	NA	NA	NA	NA	NA	NA	NA	NA	NA
	Sep	NA	NA	NA	NA	NA	NA	NA	NA	NA
**Reclaimed water**	May A	1.11	18.2	120.5	808	7.95	275.5	16.2	10.99	116.05
	May B	3.84	28.4	83.2	1.3	7.76	68.8	36.3	41.4	807.39
	Jun A	2.53	19.8	14.8	874	7.17	199.8	3.4	0.7	67.04
	Jun B	1.01	23.75	22.57	951	6.81	− 158.3	20.7	2.59	78.46
	Jul	0.03	25.5	12.83	1083	6.9	−161.5	−1.8	0.33	47.08
	Aug	8.12	23.85	100.6	821.5	6.85	246.95	3.55	1	16.63
	Aug B	0.98	22.45	29.9	859	NA	NA	10.8	2.13	36.54
	Sep	0	17.85	31.1	696.5	7.52	222.1	7.5	5.35	1703.39
**Tidal brackish water**	May A	0.89	20.338	58.9	19,815.4	6.74	275.5	2.86	12.89	8795.25
	May B	4.01	24.139	31.5	2494.3	6.22	184.7	3.67	6.5	852.57
	Jun A	7	20.433	32.9	2438.9	6.48	189.7	8.19	6.5	1016.76
	Jun B	2.36	27.37	27.1	12,140.4	6.76	156.2	4.32	22.34	4656.02
	Jul	0.09	27.09	32.6	20,524	6.77	257	−0.315	34.03	9823.28
	Aug	4.72	29.17	29.4	11,771	7	167.3	5.12	30.58	6507.61
	Aug B	0.64	28.17	26.1	21,921	7.09	129.3	1.93	41.53	13,797.6
	Sep	4.08	23.26	23.9	20,933.2	7.27	150.5	2.26	25.39	14,127.69
**Pond water**	May A	1.79	19.9	111.9	145.4	7.99	234	1.6	0.25	7.22
	May B	4.65	22.9	41.47	125.2	6.89	114.95	15	0.67	2.61
	Jun A	4.03	18.6	41.05	99.03	6.7	177.85	11.6	1.03	3.99
	Jun B	1.43	27.3	111.8	104	7.13	151.8	11.17	0.43	0.73
	Jul	0	27.95	103.5	133.9	7.24	100.2	0.97	0.09	0.12
	Aug	4.51	27.8	164	85.625	7.92	119.9	2.8	0.23	2.76
	Aug B	0.48	27.15	96.7	97.85	7.47	136.1	4.6	0.22	0
	Sep	3.71	23.1	103.8	114.7	7.06	110.6	−3.1	0.23	0.4

### Culture data

After 3 rounds of isolation and purification, 87 sucrose-positive (yellow) and 28 sucrose-negative (green) purified colonies were obtained from Thiosulfate-citrate-bile salts-sucrose (TCBS) agar resulting in a total of 115 presumptive *Vibrio* isolates from the four sites during the entire sampling period. Of the 115 presumptive *Vibrio* isolates, 29 (25.22%) isolates were confirmed via multiplex PCR as vibrios: 17 (14.8%) were confirmed as *V. cholerae*; 11 (9.6%) were confirmed as *V. parahaemolyticus*; and 1 (0.87%) was confirmed as *V. vulnificus.* The *V. cholerae* isolates were predominantly obtained from tidal brackish water (13 isolates), followed by non-tidal freshwater (2 isolates) and reclaimed water (2 isolate). *V. parahaemolyticus* isolates were also predominantly obtained from tidal brackish water (9 isolates), and two isolates were obtained from reclaimed water. The one *V. vulnificus* isolate was recovered from reclaimed water.

Table 2 describes the observed prevalence of confirmed *Vibrio* spp. by sample type and enrichment media. APW and EPW were similarly effective at detecting *Vibrio* spp. in reclaimed water samples. However, APW seemed to be more effective than EPW in the detection of *V. parahaemolyticus* and *V. cholerae* in tidal brackish water samples.

**Table 2:** Prevalence of *V. parahaemolyticus, V. cholerae* and *V. vulnificus* in nontraditional irrigation water samples processed using culture methods with two different types of enrichment media

Number of Samples (%) Positive for ***Vibrio*** spp.						
Sampling sites	***V. parahaemolyticus***		***V. cholerae***		***V. vulnificus***	
	APW	EPW	APW	EPW	APW	EPW
Reclaimed water (*n* = 8)	1 (12.5%)	1 (12.5%)	1 (12.5%)	1 (12.5%)	1 (12.5%)	0
Tidal brackish water (*n* = 8)	3 (37.5%)	2 (25%)	6 (50%)	3 (37.5%)	0	0
Nontidal freshwater (*n* = 6)	0	0	0	1 (16.67%)	0	0
Pond water (*n* = 8)	0	0	0	0	0	0

*APW* Alkaline peptone water enrichment. *EPW* Estuarine peptone water enrichment

### 16S rRNA sequencing dataset

Extracted DNA from a total of 180 sub-samples as described in Table 3 was PCR- amplified for the 16S rRNA gene and sequenced using the Illumina HiSeq platform. 6,302,683 sequences were generated in total across all samples, and clustered into 17,237 operational taxonomic units (OTUs). Across all samples, the minimum number of reads was 357 and the maximum was 99,944, with an average number of sequences per sample of 35,014.91 (+/− 14,897.3 SD). A Goods estimate coverage of 0.90 was calculated for all samples. Three control samples that were not enriched (1 reclaimed water, 1 pond water and 1 non-tidal freshwater creek sample) had a Good’s estimate coverage < 0.90 and were, therefore, removed to ensure appropriate read coverage across all samples analyzed downstream (Supplementary Figure S1). After data cleanup (removing OTUs with less than 10 reads), the total number of sequences used in downstream analyses was 6,020,192 from 177 samples (*n* = 47 pond water, n = 47 reclaimed water, *n* = 35 non-tidal fresh water creek and *n* = 48 tidal brackish water samples), clustered into 7298 OTUs with a minimum number of 2901 reads and a maximum of 99,857 reads.

**Table 3:** Summary of total sub-samples tested using our coupled labeling and sequencing approach by water type, BrdU treatment and enrichment method

Sampling site	BrdU treatment			No BrdU treatment			Total (N)
	APW	EPW	No enrichment	APW	EPW	No enrichment	
Pond water	8	8	8	8	8	8	48
Reclaimed water	8	8	8	8	8	8	48
Non-tidal freshwater	6	6	6	6	6	6	36
Tidal brackish water	8	8	8	8	8	8	48
Total (N)							180

*APW* Alkaline peptone water enrichment. *EPW* Estuarine peptone water enrichment

### Alpha and beta diversity

**Fig. 1:**
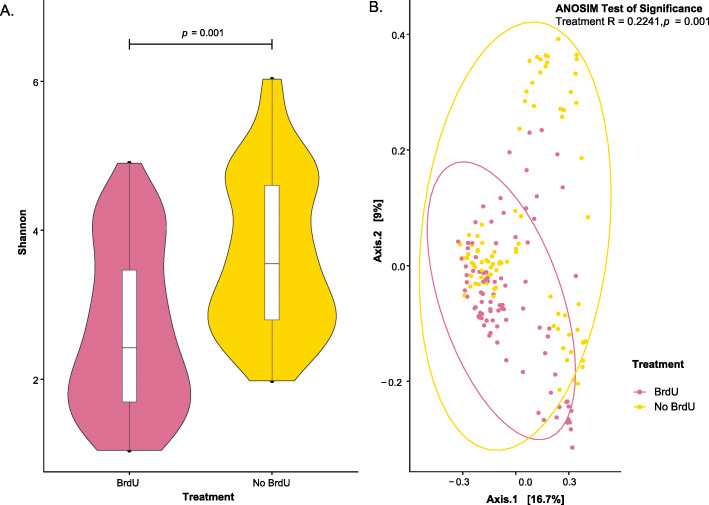
**A** Box plot of alpha diversity (Shannon Index) across all samples on rarefied data to minimum sampling depth. Alpha diversity of BrdU-treated samples represents the diversity observed in the metabolically-active fraction of bacterial communities present in each sample. Pink represents BrdU-treated samples and yellow represents non-BrdU treated samples. **B** PCoA analysis of Bray Curtis computed distances between BrdU- and non-BrdU-treated water samples. Solid colored ellipses are drawn at 95% confidence intervals by treatment type of the water samples

Alpha diversity metrics (Shannon diversity) were calculated on both rarefied (after down-sampling each sample to 2901) and non-rarefied data (data not shown) to avoid sequence coverage issues. Since no differences were observed between the rarefied and non-rarefied analysis, we only presented alpha-diversity analysis performed on the rarefied dataset in Fig. 1A. Irrespective of sampling site/water type, the alpha diversity of BrdU-treated samples was significantly lower (*p* < 0.001) when compared to non-BrdU treated samples (Fig. 1A).

Principal coordinate analysis using Bray Curtis distances was implemented to quantify the inter-sample diversity (beta diversity). The analysis revealed that bacterial profiles associated with BrdU-treated samples were similar to the non-BrdU treated samples and showed slight variation by treatment (ANOSIM, R value =0.2241, *p* = 0.001) (Fig. 1B).

### Taxonomic analysis

The top five bacterial phyla identified across all sampling sites irrespective of treatments and enrichments were *Proteobacteria*, *Firmicutes*, *Bacteroidetes, Actinobacteria*, and *Fusobacteria*. The most predominant phyla with an average relative abundance of 44.55% (+/− 0.21) was *Proteobacteria*, followed by *Firmicutes* that had an average relative abundance of 24.40% (+/− 0.26). *Bacteroidetes, Actinobacteria* and *Fusobacteria* had an average relative abundance of 15.58% (+/− 0.18), 11.45% (+/− 0.13) and 0.96% (+/− 0.04) respectively.

**Fig. 2:**
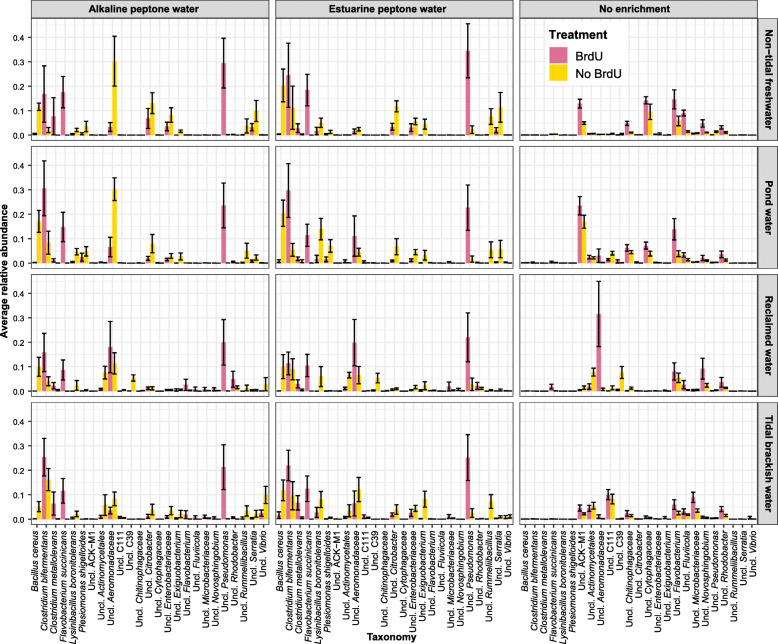
Taxonomic profiles of the top 25 bacteria detected in pond water, tidal brackish water, reclaimed water and non-tidal freshwater derived from 16S rRNA sequencing data. Pink represents BrdU-treated samples and yellow represents non-BrdU treated samples

In total, 2205 (30%) OTUs were assigned to the genus level of which only 351 (5%) could be identified to the species level. The top 25 bacterial taxa across all sampling sites, enrichments and treatments were *Clostridium bifermentans,* unclassified *Aeromonadaceae, Pseudomonas, Bacillus cereus, Flavobacterum succinicans, Citrobacter*, unclassified ACK-M1*, Flavobacterium,* unclassified *Actinomycetales, Lysinibacillus boronitolerans,* unclassified *Enterobacteriaceae, Serratia,* unclassified *Cytophagaceae, Rummeliibacillus, Clostridium metallolevans, Rhodobacter, unclassified C111, Exiguobacterium, Fluviicola, Novosphingobium, Plesiomonas shigelloides,* unclassified *Chitinophagaceae*, unclassified *Microbacteriaceae*, unclassified C39 and *Vibrio* (Fig. 2)*.*

**Fig. 3:**
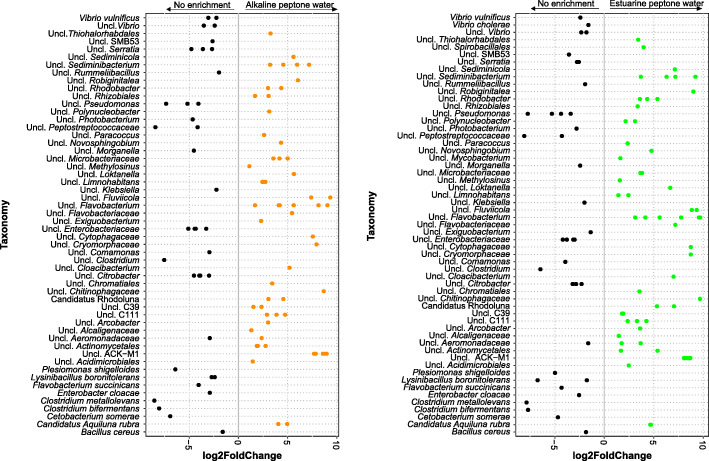
Differential abundances of bacterial genera that were statistically significant (*p* < 0.05) in BrdU treated samples between enrichments: no enrichment versus alkaline peptone water (APW), and no enrichment versus estuarine peptone water (EPW). A positive log2-fold change denotes a bacterial taxonomy that is significantly higher in either enrichments (APW or EPW), while a negative log2-fold change indicates a bacterial taxonomy that is significantly higher in no enrichment BrdU-treated samples. The grey line and arrows highlight the conversion in log2-fold change from negative to positive values

Differential abundance analysis was performed to identify bacterial genera that were significantly different (*p* < 0.05) between enrichments (APW versus no enrichment and EPW versus no enrichment) in all BrdU treated samples (Fig. 3). *Vibrio cholerae, Vibrio vulnificus*, *Clostridium metallolevans, L. boronitolerans*, *F. succinicans, Enterobacter cloacae*, *Cetobacterium somerae*, *B. cereus, P. shigelloides* and *C. bifermentans* were found at a significantly higher abundance in BrdU-treated, non-enriched samples. Additionally, all BrdU-treated enriched (APW and EPW) samples were characterized by a higher relative abundance of Candidatus *Aquiluna rubra* (*Actinobacteria*).

### *Vibrio* taxonomy

**Fig. 4:**
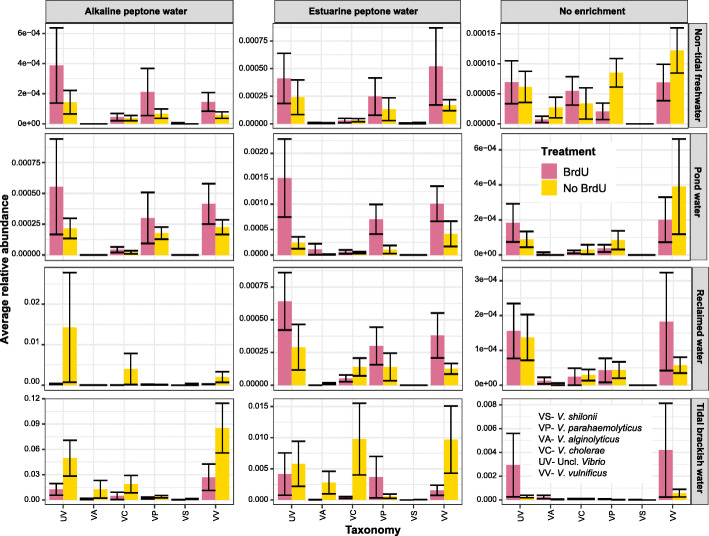
Average relative abundance of *Vibrio* species in pond water, tidal brackish water, reclaimed water and non-tidal freshwater in different enrichments (APW, EPW and no enrichment). *Vibrio* species abbreviation: VS = *V. shilonii*, VP = *V. parahaemolyticus*, VA = *V. aestaurinus*, VC = *V. cholerae*, UV = Unclassified *Vibrio* and VV- *V. vulnificus*

Irrespective of sampling site, treatments and enrichments, we were able to observe vibrios in the 16S rRNA sequencing data of all samples at a low relative abundance (Fig. 4). Some of the species observed were *V. cholerae*, *V. vulnificus*, *V. parahaemolyticus*, *V. aestaurinus* and *V. shilonii*. Among the sampling sites, tidal brackish water samples were characterized by the highest relative abundance of vibrios*,* followed by reclaimed water samples. Additionally, in the non-enriched BrdU-treated tidal brackish water samples, we observed *Vibrio* spp., indicating the detection of metabolically-active, viable vibrios*,* including *V. vulnificus,* without the aid of enrichment techniques. In non-tidal freshwater, reclaimed water and pond water samples, a higher relative abundance of metabolically-active vibrios coincided with the use of enrichment techniques.

Additionally, we observed several bacterial taxa that were correlated with the presence of *Vibrio* (Supplementary Fig. S3). The taxa observed when *Vibrio* were present in the water samples were Uncl. *Pseudomonas*, Uncl. *Microbacteriaceae*, Uncl. c111*, Plesiomonas shigelloides*, *Flavobacterium succinicans, Clostridium metallovens* and *Clostridium bifermentans.*

### Comparison between culture-based and 16S rRNA *Vibrio* spp. detection

Table 4 provides a comparison of *Vibrio* spp. detection using culture-based versus 16S rRNA sequencing approaches with and without BrdU labeling. Interestingly, 16S rRNA sequencing frequently detected *V. aesturianus, V. cholerae, V. parahaemolyticus, V. shilonii,* and *V. vulnificus* across multiple water sample types when our culture-based approach was unable to detect these organisms (Table 4). Most importantly, 16S rRNA sequencing of BrdU labeled samples resulted in *Vibrio* spp. detection even when our culture-based methods resulted in negative samples (Table 4). This suggests that our coupled labeling and sequencing approach was able to detect metabolically-active *Vibrio* spp. that may have been present in a VBNC state.

**Table 4:** Detection of *Vibrio* spp. in nontraditional irrigation water samples using culture-based methods versus 16S rRNA sequencing with and without BrdU labeling

Sampling sites/ detection methods	APW			EPW			No enrichment	
	Culture	16S-BrdU	16S- No BrdU	Culture	16S-BrdU	16S- No BrdU	16S-BrdU	16S- No BrdU
***V. aesturianus***								
Reclaimed water (*n* = 48)	Absent	0.002	0.003	Absent	0	0.001	0.001	0.0003
Tidal brackish water (*n* = 48)	Absent	0.1	1.3	Absent	0	0.3	0.02	0
Nontidal freshwater (*n* = 36)	Absent	0	0	Absent	0.0008	0.0007	0.0007	0.003
Pond water (*n* = 48)	Absent	0	0	Absent	0.013	0	0	0
***V. cholerae***								
Reclaimed water (*n* = 48)	Present	0.004	0.4	Present	0.005	0.014	0.002	0.003
Tidal brackish water (*n* = 48)	Present	0.5	1.9	Present	0.04	1	0.009	0.009
Nontidal freshwater (*n* = 36)	Absent	0.004	0.004	Present	0.003	0.003	0.005	0.003
Pond water (*n* = 48)	Absent	0.004	0.002	Absent	0.006	0.005	0.002	0.003
***V. parahaemolyticus***								
Reclaimed water (*n* = 48)	Present	0.01	0.01	Present	0.03	0.013	0.004	0.004
Tidal brackish water (*n* = 48)	Present	0.24	0.4	Present	0.37	0.06	0.007	0.002
Nontidal freshwater (*n* = 36)	Absent	0.021	0.007	Absent	0.024	0.013	0.002	0.008
Pond water (*n* = 48)	Absent	0.03	0.0001	Absent	0.07	0.01	0.004	0.008
***V. shilonii***								
Reclaimed water (*n* = 48)	Absent	0	0.01	Absent	0	0	0	0
Tidal brackish water (*n* = 48)	Absent	0.02	0.06	Absent	0	0.0025	0.001	0
Nontidal freshwater (*n* = 36)	Absent	0.0004	0	Absent	0.0004	0.0006	0	0
Pond water (*n* = 48)	Absent	0	0	Absent	0	0	0	0
***V. vulnificus***								
Reclaimed water (*n* = 48)	Present	0.007	0.07	Absent	0.01	0.004	0.009	0.002
Tidal brackish water (*n* = 48)	Absent	0.9	2.84	Absent	0.05	0.32	0.14	0.02
Nontidal freshwater (*n* = 36)	Absent	0.005	0.002	Absent	0.02	0.006	0.002	0.009
Pond water (*n* = 48)	Absent	0.015	0.006	Absent	0.03	0.01	0.007	0.013

## Discussion

The nontraditional irrigation water sources tested in our study harbored diverse bacterial communities (some of which are of concern to public health), and hence, these water sources would likely require treatment prior to their use for food crop irrigation. Specifically, we were able to detect the presence of *Vibrio* spp. across all sampling sites. Our culture-based methods were only able to detect *Vibrio* spp. at three of the sampling sites while our coupled BrdU-labeling and 16S rRNA sequencing approach revealed the presence of vibrios at all four sampling sites (Table 4). Most interestingly, our novel approach of coupling BrdU labeling with 16S rRNA sequencing was able to detect metabolically-active vibrios in water samples that were negative based on our culture results, suggesting that our approach can detect metabolically-active *Vibrio* spp. that may be present in a VBNC state. We also could detect metabolically-active vibrios in non-enriched samples from all sampling sites, indicating that our coupled method could be helpful in quickly detecting VBNC vibrios without the use of a time-consuming enrichment step.

The bacterial genus *Vibrio* is ubiquitous and widely distributed in aquatic environments from brackish water to deep seawater, worldwide [19]. These bacteria have also been found in different surface waters [37, 52, 65] and reclaimed water [25, 52, 53]. Most *Vibrio*-associated illnesses have been associated with either foodborne infections caused by the consumption of raw or undercooked seafood or wound infections acquired during aquatic activities in coastal or estuarine waters [19]. Very rarely, instances of *Vibrio* outbreaks associated with the consumption of raw vegetables have been reported [9, 28, 63, 76]. For instance *Vibrio* contamination of vegetables irrigated with partially-treated municipal wastewater in Varanasi, India was reported [63]. Additionally, *V. cholerae* O1 was detected in vegetables that were irrigated using wastewater and stabilization ponds in Tanzania [28]. The prevalence of *V. parahaemolyticus* in raw salad vegetables at the retail level was also observed in Malaysia [76]. Nevertheless, to date no *Vibrio* outbreaks in the U.S. have been associated with the consumption of fresh produce.

Traditionally, pre-enrichment assays (alkaline peptone water) prior to culturing on selective media (thiosulfate-citrate-bile salts-sucrose agar) have been commonly used to improve the detection of pathogenic vibrios from environmental sources [31]. However, these methods are labor intensive, costly and fail to capture VBNC vibrios*,* thereby resulting in an underestimation of the prevalence of vibrios in the environment. With advances in molecular detection methods (e.g. multiplex *Vibrio*-specific PCRs) there has been an increased frequency in the detection of vibrios [38]. Yet, viability of the detected organisms is questionable as molecular methods rely on the detection of DNA that can persist in the environment long after bacteria have died. To address this issue, previous studies have employed DNA intercalating dyes such as ethidium monoazide (EMA) and propidium monoazide (PMA) to estimate the total number of viable cells in environmental samples [5, 6, 41]. Recently, Cao et al. [6] was able to detect VBNC *V. parahaemolyticus* in shrimp samples by utilizing PMA dyes [6]. Though this intercalating dye looks promising and is being widely used to detect metabolically-active bacteria, a recent study by Li et al. [40] found that upon comparing DNA-, PMA- and RNA-based 16S rRNA sequencing, the PMA-based approaches tend to overestimate the live or metabolically-active bacterial population when compared to RNA-based methods.

Prior to the present study, there has been no study using BrdU in tandem with sequencing to detect metabolically-active environmental vibrios, but several studies have used BrdU to detect other metabolically-active bacteria in different environmental samples [44, 46, 75], and improve understanding of cell proliferation with regard to adult neurogenesis [30, 74]. Additionally, BrdU coupled with qPCR has been extensively used to detect persistent fecal bacteria in sewage effluent [79], psychrotolerant bacteria in polluted sea sediments [20] and the impact of mycorrhizal fungi on bacterial communities in soil [2]. Recently, our group coupled BrdU with sequencing techniques to detect metabolically-active bacterial communities in pond and reclaimed water in the mid-Atlantic region [44]. In the present study, our culture-based method was unable to detect *Vibrio* spp. in any pond water samples, while our 16S rRNA sequencing data (generated from both BrdU treated and non-treated samples) identified *V. cholerae*, *V. parahaemolyticus* and *V. vulnificus* in enriched and non-enriched pond samples (Table 4), indicating the detection of likely VBNC vibrios that the culture-based method could not detect. Also, in all non-enriched BrdU-treated water samples we were able to detect *Vibrio* spp. (Table 4, Fig. 4), suggesting that our coupled BrdU-labeling and sequencing approach could potentially replace laborious enrichment approaches. Moreover, we were able to detect the presence of other *Vibrio*’s including *V. shilloni* (coral pathogen) [39] and *V. aesturianus* (oyster pathogen) [42] using our coupled labeling and sequencing approach when our culture-based approach was unable to detect these species (Table 4).

Besides *Vibrio* spp., our findings also revealed the presence of other human bacterial pathogens in BrdU-treated enriched samples including *C. bifermentans*, *B. cereus*, *P. shigelloides*, and *E. cloacae* (Fig. 2). *C. bifermentans* is a Gram-positive bacillus known to occur in water, soil, sewage [51], sludge and animal feces [80] and is a rare human pathogen. Unlike other *Clostridium* species like *C. botulinum* and *C. perfringes* (common causes of foodborne illness) [22, 24], *C. bifermentans* infections have been reported rarely (14 cases to date), none of which have been associated with food-borne illness [26].

In contrast, *B. cereus,* another Gram-positive, aerobic-to-facultative, spore-forming rod that is widespread in nature, has been frequently isolated from soil and growing plants [4] and has been associated with food-associated illness [8, 72]. Outbreaks of *B. cereus* have been reported as a result of consumption of contaminated vegetable sprouts [61] and refried beans served at a fast food restaurant chain in upstate New York [8]. In addition, [78] characterized *B. cereus* isolates from nearly 56 samples of fresh vegetables (peppers, cucumbers, tomatoes, carrots, zucchini, garlic and onions) and in refrigerated, minimally processed foods that included these vegetables as the ingredients [78]. The presence of these organisms in refrigerated, minimally processed foods demonstrates their persistence through food processing methods.

With regard to *P. shigelloides,* a total of 11 outbreaks have been reported worldwide from 1961 to 2003, of which four outbreaks occurred in the US [34]. Sources of these outbreaks were mainly contaminated shellfish, fish, meat products, and contaminated water sources (tap, well and freshwater) [34]. The common environmental reservoirs for these bacteria include freshwater ecosystems and estuaries [34]. Indirect contamination with *P. shigelloides* after major natural aquatic disasters has also been reported [68]. For instance, after the 2004 tsunami episode in India, *P. shigelloides,* along with pathogenic vibrios, *Aeromonas* and *Plesiomonas,* were isolated from hand pumps and wells in several communities [35].

*E. cloacae,* another pathogen detected using our coupled BrdU labeling and sequencing method, has been reported as an opportunistic and multi-drug resistant bacterial pathogen involved in hospital associated outbreaks between 1993 and 2003 in Europe [18]. *E. cloacae* is ubiquitous in terrestrial and aquatic environments and occurs as a commensal in the intestines of humans and animals, making it a perfect candidate for transfer from irrigated produce to humans. *E. cloacae* have been isolated from ready-to-eat salads served in a primary school in Valencia city [23] and from vegetables irrigated with untreated wastewater in Morocco [33].

Strengths of this study include the sample size, diversity of water sample types, thorough statistical analysis of our data, our ability to have a head-to-head comparison between traditional culture-based methods and our coupled labeling/sequencing method, and the ability to ultimately differentiate between live/metabolically-active and relic/dead bacterial communities using our novel method. Additionally, we observed the co-occurrence of other bacterial taxa when *Vibrio* were present in the tested water samples, indicating that these taxa could potentially serve as proxies for *Vibrio* presence in the environment. This is an interesting area for future research.

Like all 16S rRNA-based sequencing techniques, our study limitations include PCR amplification biases, limited ability to assign species-level classifications (limitations of the currently-available databases), and limited ability to distinguish various *Vibrio* species among the *Vibrionaceae* family (due to similar 16S rRNA sequences among the different species). In terms of the BrdU labeling method, it is possible that the 24 h incubation period that we utilized (for both BrdU-labeled and non-labeled samples) may have influenced the bacterial communities observed in our study; however, any changes would have occurred in both BrdU-labeled and non-labeled samples similarly.

Despite these limitations, our findings demonstrate that coupling BrdU-labeling with 16S rRNA sequencing enables the detection of metabolically-active *Vibrio* spp. that may be present in water samples in a VBNC state. In addition, we showed that our coupled labeling and sequencing approach can detect vibrios in non-enriched BrdU-labeled samples, indicating that the use of this method could replace laborious enrichment steps, shortening time to detection. Finally, our novel method could be used in the future to quickly detect other food- and waterborne pathogens, including *Salmonella* and *Campylobacter,* that may be present in a VBNC state and can be difficult to detect using conventional culture-based methods.

## Supplementary Information

**Additional file 1: Supplementary Fig. 1.** Good’s coverage among the different water sampling sites.

**Additional file 2: Supplementary Fig. 2.** Description of the sample processing and analysis approach.

**Additional file 3: Supplementary Fig. 3.** Co-occurrence of bacterial taxa in the presence and absence of *Vibrio,* visualized by a chord plot.

## Data Availability

The datasets generated and/or analyzed during the current study are deposited in the NCBI BioProject database under BioProject accession number PRJNA473136.
