# Supplementary figures and images for: Coupled DNA-labeling and sequencing approach enables the detection of viable-but-non-culturable Vibrio spp. in irrigation water sources in the Chesapeake Bay watershed
Source: Environ Microbiome. 2021 Jun 22;16:13. doi: 10.1186/s40793-021-00382-1 (PMC8218497; doi:10.1186/s40793-021-00382-1)

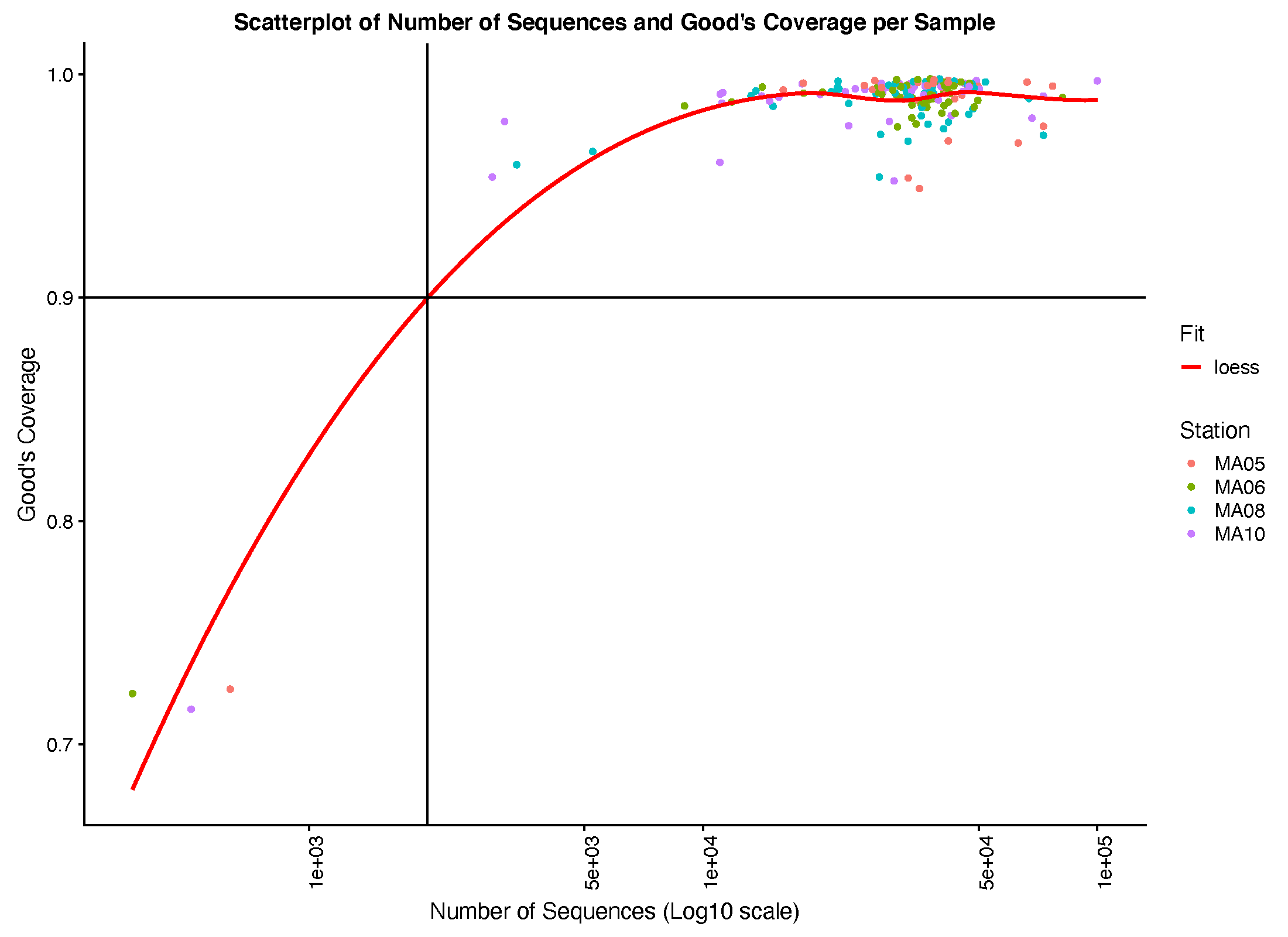

Supplement: Supplementary file 1 — Additional file 1: Supplementary Fig. 1. Good’s coverage among the different water sampling sites. [file 40793_2021_382_MOESM1_ESM.tiff]

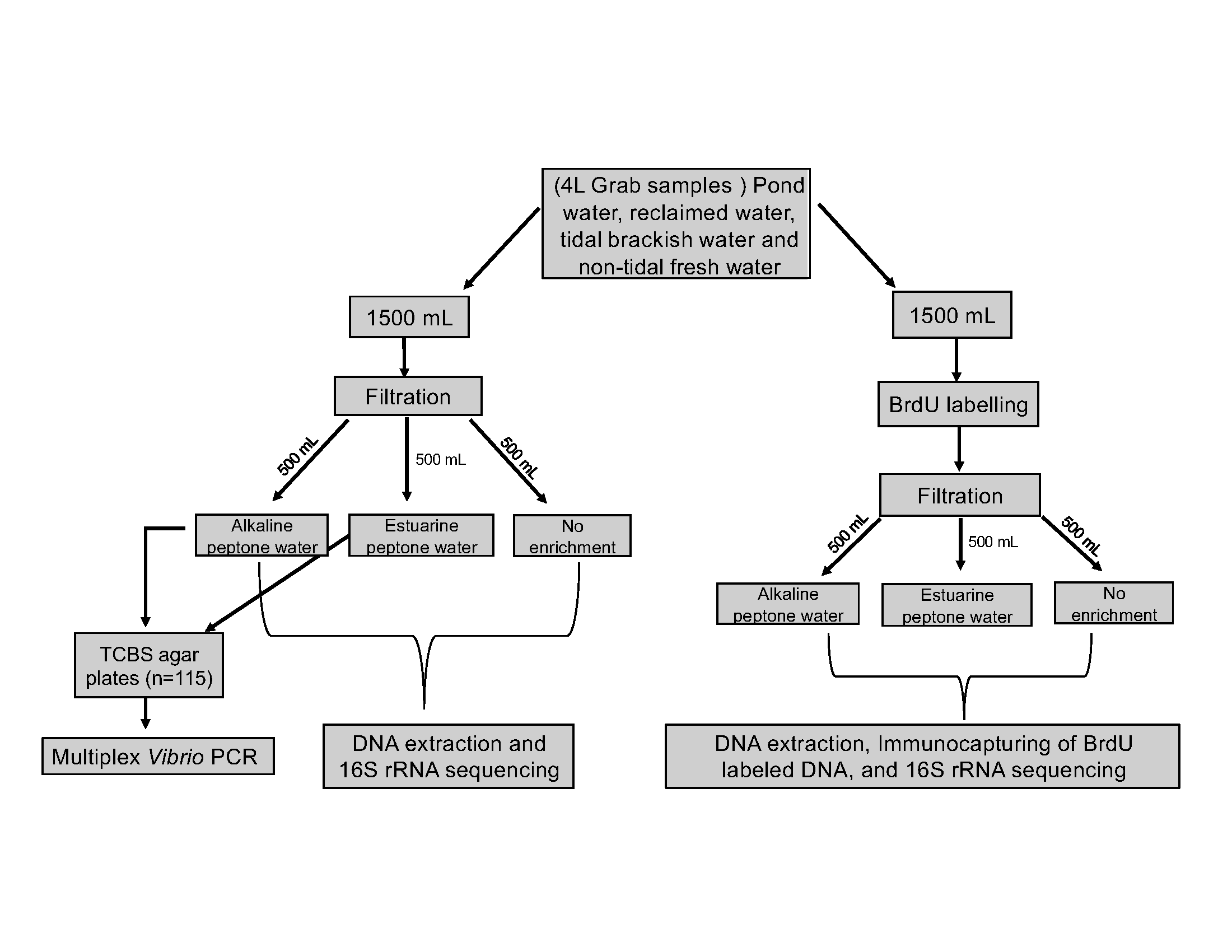

Supplement: Supplementary file 2 — Additional file 2: Supplementary Fig. 2. Description of the sample processing and analysis approach. [file 40793_2021_382_MOESM2_ESM.tiff]

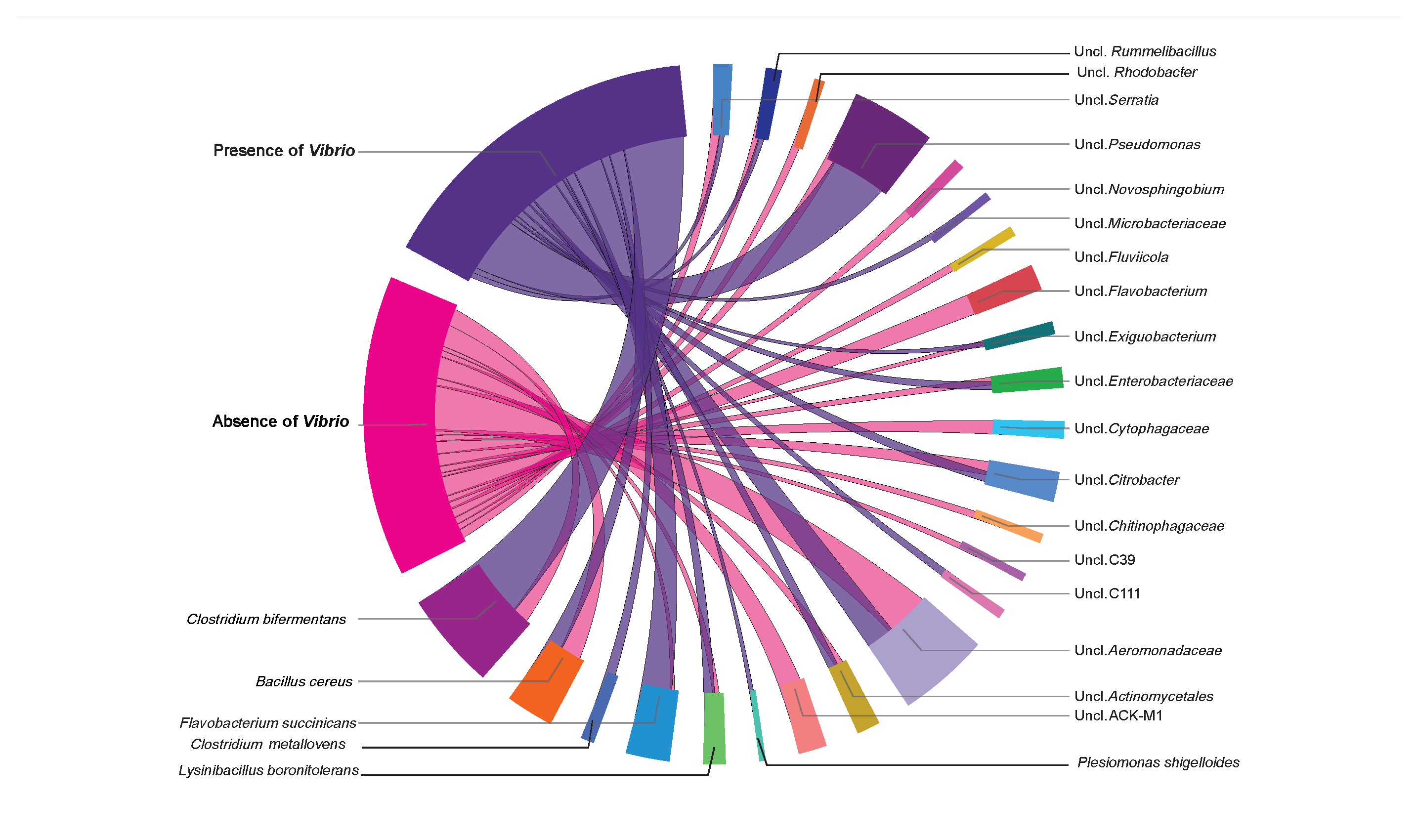

Supplement: Supplementary file 3 — Additional file 3: Supplementary Fig. 3. Co-occurrence of bacterial taxa in the presence and absence of Vibrio, visualized by a chord plot. [file 40793_2021_382_MOESM3_ESM.tiff]
